# In the Eye of the Beholder: Owner Preferences for Variations in Cats’ Appearances with Specific Focus on Skull Morphology

**DOI:** 10.3390/ani8020030

**Published:** 2018-02-20

**Authors:** Mark J. Farnworth, Rowena M. A. Packer, Lorena Sordo, Ruoning Chen, Sarah M. A. Caney, Danièlle A. Gunn-Moore

**Affiliations:** 1 School of Animal, Rural and Environmental Sciences, Nottingham Trent University, Southwell, Nottinghamshire NG25 0QF, UK; 2 Department of Clinical Science and Services, Royal Veterinary College, Hatfield, Hertfordshire AL9 7TA, UK; rpacker@rvc.ac.uk; 3 The Royal (Dick) School of Veterinary Studies and The Roslin Institute, University of Edinburgh, Edinburgh EH25 9RG, UK; Lorena.sordo@ed.ac.uk (L.S.); ruoningchen@163.com (R.C.); Danielle.Gunn-Moore@ed.ac.uk (D.A.G.-M.); 4 Vet Professionals Ltd, Midlothian Innovation Centre, Pentlandfield, Roslin, Midlothian EH25 9RE, UK; Sarah@vetprofessionals.com

**Keywords:** brachycephaly, cat, dolichocephaly, pedigree, preference, ownership

## Abstract

**Simple Summary:**

Recently, there has been an increase in popularity of cats with different skull shapes, including shortened or lengthened muzzles. Skull shape, like other physical features, may affect human preferences; however, it is also more likely to have an impact on the welfare of the cat. We asked people to score their preference for 15 pictures of cats across two surveys. Extreme face shapes (those that were very short or very long) were least preferred. Short-faced cats were less popular amongst cat owners from animal related jobs as opposed to other people. Respondents that had a short or long-faced cat preferred cats with the same skull shape, but also had lower preferences for the opposite skull shape. Respondents from Asia, as compared to those from elsewhere, gave higher preference scores to both long and short-faced cats. Amongst the other features, green eyes, a ginger coat color and medium length coat were most preferred, although the ability to draw conclusions around these features is limited, given they are not necessarily independent of skull shape. This study provides the first evidence that preferences for cat breeds, and their associated skull morphologies, are driven by both culture and owner experience. This information may inform future research concerning the preferences of cat owners.

**Abstract:**

Changes in the popularity of cat breeds are largely driven by human perceptions of, and selection for, phenotypic traits including skull morphology. The popularity of breeds with altered skull shapes appears to be increasing, and owner preferences are an important part of this dynamic. This study sought to establish how and why a range of phenotypic attributes, including skull shape, affect preferences shown by cat owners. Two questionnaires were distributed on-line to cat owners who were asked to rate preferences for pictures of cats on a 0–10 scale. Veterinarian consensus established the skull types of the cats pictured (i.e., level of brachycephaly (BC) or dolichocephaly (DC)). Preferences were then explored relative to cat skull type, coat and eye color, and coat length. Generalized estimating equations identified relationships between physical characteristics and respondent ratings. Further sub-analyses explored effects of respondents’ occupation, location and previous cat ownership on rating scores. Overall, cats with extreme changes in skull morphology (both BC and DC) were significantly less preferred than mesocephalic cats. Green eyes, ginger coat color and medium length coat were most preferred. Current owners of a BC or DC pure bred cat showed significantly greater preference for cats with similar features and significantly lower preference for the opposite extreme. Respondents from Asia were significantly more likely to prefer both BC and DC cats as compared to respondents from other locations. Finally, those in an animal care profession, as compared to other professions, provided a significantly lower preference rating for BC cats but not for DC cats. This work, despite the acknowledged limitations, provides preliminary evidence that preferences for cat breeds, and their associated skull morphologies, are driven by both cultural and experiential parameters. This information may allow for better targeting of educational materials concerning cat breeds.

## 1. Introduction

Since their self-domestication some 10,000 years ago [1], cats have undergone numerous genotypic [2] and phenotypic [3,4] changes as a result of selective breeding. Despite the relatively recent proliferation of fancy cat breeds, the genotypic changes that underlie breed-specific features are now able to be distinguished [5], suggesting substantial segregation of genotypes at the breed level. Historically, the proportion of the owned cat population in the United Kingdom reported as pedigree or purebred is between 8% [6] and 11% [7], with around 3% of cats registered with a veterinarian being Persian [6]. Demographics of the cat population will, in part, be driven by human preferences for specific conformational traits and the intrinsic value placed upon them [8,9].

Numerous factors may influence an owner’s choice of a companion animal. During adoption, evidence suggests that problem behaviors in cats and dogs significantly reduce their likelihood of rehoming [10,11]. Older cats have a longer duration of stay within the shelter environment [11] and dark coat coloration both reduces adoption likelihood [10] and increases length of stay [11]. With the advent of social media, the opportunity to assess animals through static photographs, and without direct interaction has increased, despite evidence that a cat’s human directed behaviors (e.g., rubbing) may play a greater role in speed of adoption than more subtle facial characteristics and movements [12].

Studies suggest that the popularity of different dog breeds is influenced by media exposure, even though some of today’s most commonly used breeds experience health problems associated with their appearance [13,14]. Recent research indicates that the choice to own a brachycephalic (BC) dog breed is driven by its appearance, which is often prioritized above breed health [15]. This effect may also apply to cats, with BC breeds (Exotic Shorthair; British Shorthair; Persian; Scottish Fold) and dolichocephalic breeds (DC) (Sphynx; Abyssinian) now comprising six of the top 10 breed registrations in the United States of America [16]. Much of the recent literature concerning owner reports of, and attitudes towards, the impacts of changes in skull morphology focuses on BC dog breeds [17,18], with limited evidence on the health implications of skull shape in cats and attitudes towards them. However, there is growing evidence that brachycephalism in cats is directly associated with disorders that impact respiratory [19,20,21], ophthalmic [22,23], endocrine [24] and neurological [25,26] health. Many of these issues require corrective surgical intervention to improve the welfare of the individuals affected [27,28,29,30]. Those conditions that cannot be alleviated may result in a reduced quality of life. However, such conditions do not necessarily affect longevity equally, skull shape is only once a genotypic component of any given breed, and longevity will be determined by a number of heritable and environmental factors. For example, Persian cats generally experience lives of equal or greater length to crossbreeds (median 14.1 years and 14 years respectively), whilst British Shorthairs’ lifespans are comparatively reduced (median 11.8 years) [31]. The growing body of evidence concerning the impacts of brachycephalism has prompted debate within the scientific literature as to whether the breeding of BC cats should be reconsidered [32,33]. In contrast, dolichocephaly has remained relatively underexplored in terms of direct consequences arising from craniofacial morphology. In DC cats, evidence of breed-related issues appear to be largely associated with characteristics other than skull shape [34]. There is some evidence of degenerative ocular disorders in DC cats that are related to breeding rather than skull conformation [35]. Similar to BC cats, the impacts of breed selection on longevity of DC cats are equivocal, with Siamese cats living longer (median 14.2 years), and Abyssinians substantially shorter lives (median 10 years) as compared to crossbreeds (median 14 years) [31].

How preferences for cat breeds are formed, especially those cats with extreme skull shape, is little understood. The objectives of this research were to explore:

(i) The relative popularity of phenotypic features in static images of cats

(ii) How respondent-related factors impacted their preference for cats’ phenotypic features, with particular focus on skull shape.

## 2. Materials and Methods

### 2.1. Recruitment of Participants and Questions Asked

Participants were recruited on two occasions, as part of a wider survey of the health and lifestyle of the respondent’s cat, using an all-available sampling methodology. The surveys targeted the general public and professionals in animal-careers as well as owners of pedigree cats. All respondents were cat owners and aged 18 years or over. Both iterations of the questionnaire were ethically approved by the Human Ethics Research Committee of Edinburgh University. Surveys were disseminated twice based on the target audience and language. Survey two also contained additional questions about eye health, which were not considered in this manuscript. The first iteration of the survey was disseminated within the UK and Asia (primarily China) between February and July 2015, and the second primarily within the UK and South America (Mexico, Argentina, Peru, Uruguay, Chile and Ecuador) between February and July 2016. The English questionnaire was translated into Mandarin and Spanish depending on the primary language of the target countries. Surveys were disseminated via a link to an external site that was promoted by the Vet Professionals website (www.vetprofessionals.com), a Chinese survey website (www.wenjuan.com), International Cat Care (http://icatcare.org/) and Cats Protection (http://www.cats.org.uk/). A number of social media sites concerning cats within the target countries were also used for recruitment. The first survey asked respondents to rate photographs of nine cats on the scale 0 (“I don’t like this cat at all”)–5 (neutral)–10 (“this is my favourite type of cat”). This study was extended in a second survey, which included the original nine photographs and provided an additional six, totaling 15 photographs overall (see Table 1). Responses for the survey data used for this manuscript addressed the respondent’s gender; country of residence; breed and registration status of cat owned; and profession. No repeat responses were identified based on exploration of IP addresses.

### 2.2. Establishing Cephalic Rating for Analyses

As the 15 photographs were not standardized (e.g., angle of the cat to the camera), it was not possible to use a cephalic index based on morphological landmarks as in Farnworth et al. [19]. A veterinary panel was used to establish consensus of cephalic rating, and the images were disseminated to members of the International Society for Feline Medicine via their online forum. Respondents were asked to provide a cephalic rating for the 15 cat photographs used in the surveys. Ratings were based on the following numerical scale: 1 = extreme BC (having the shortest muzzle possible); 2 = moderate BC; 3 = mild BC; 4 = mesocephalic; 5 = mild DC; 6 = moderate DC; 7 = extreme DC (having the longest muzzle possible). The median rating for each cat was used in the analyses.

### 2.3. Assigning Coat Color for Analyses

Coat color was ascribed to broad categories based on author consensus (for images, see Table 1. Blue/grey: cats 7–10; Ginger: cats 1–3; Tabby: cats 14 & 15; White/Pale/Point: cats 4–6 and 11–13). White and “Point” types were combined as only a single white cat was included in the pictures. This process was performed because coat color can be considered an important confounding factor in the rating decision. Coat color was not controlled for during data collection and therefore the distribution of coat color types and shades was not evenly distributed amongst skull types. Therefore, the outcomes of the subsequent analyses, as they relate to coat color, should be considered with caution.

### 2.4. Statistical Analyses

Generalized estimating equations were used to evaluate associations between physical traits of the cats studied and their ratings by the surveyed cat owners, taking account of owner and cat ID as repeated measures. Univariate associations between phenotypic features (coat length, coat color, eye color and skull shape) and rating scores were analyzed using the Kruskal-Wallis test. Factors with liberal associations in univariable testing (*p*  <  0.2) were taken forward for multivariable evaluation. Model development used backwards stepwise elimination.

Sub-analyses were conducted to identify respondent-related factors associated with BC and DC preference. The effects of respondent-related variables (veterinary/animal related profession, geographical location, and skull morphology of the oldest currently owned cat) upon their ratings of either BC or DC cats were analyzed within the overall sample using either Mann-Whitney (two categories) or Kruskal-Wallis tests (over two categories). Before all analyses, variables were visually inspected for normality of distribution using histograms, and all results where *p* < 0.05 were considered significant.

## 3. Results

### 3.1. Response Rates and Descriptive Statistics

The sample totaled 1239 respondents (*n* = 411 survey one; *n* = 828 survey two). Of the total sample, 92.4% of respondents (1145/1239) were female. Given the targeted nature of the survey dissemination, 18.7% (244/1239) of respondents had worked in a veterinary or animal care related profession. In terms of geographical location, 47.1% (584/1239) were from Europe; 33.2% (412/1239) were from Asia; 11.8% from North America; 6.5% (80/1239) from South America; and 1.4% (14/1239) from Australasia.

All respondents were cat owners and, based on the target demographic of this survey, 35.6% (441/1239) reported the eldest cat they owned to be purebred. Of these 52.2% (230/441) were reported as a mesocephalic breed; 35.6% (157/441) a BC breed; and 12.2% (54/441) a DC breed. Non-pedigree crossbreed ‘moggies’ represented 64.4% (798/1239) of the population. The skull morphology of the respondent’s cat was based on the reported breed and the associated breed standard. Crossbred cats were classified unknown, due to an inability to ascribe a breed standard.

### 3.2. Cephalic Rating Consensus

The request for veterinarian opinion yielded 50 responses, of which 45 were complete. Partial responses were retained and used for those cats that had been scored, and non-responses were excluded from the data. For all cats in the sample, the median and mode were the same (see Table 1), whilst the means rating lay within the range of ± 0.4 of the median or mode with the exception of cat 7 (+ 0.6). These ratings suggest substantial consensus amongst those veterinarians that responded, outliers notwithstanding. The median rating for each cat was used in the analyses.

### 3.3. Associations between Phenotypic Features and Rating Score

**Figure 1:**
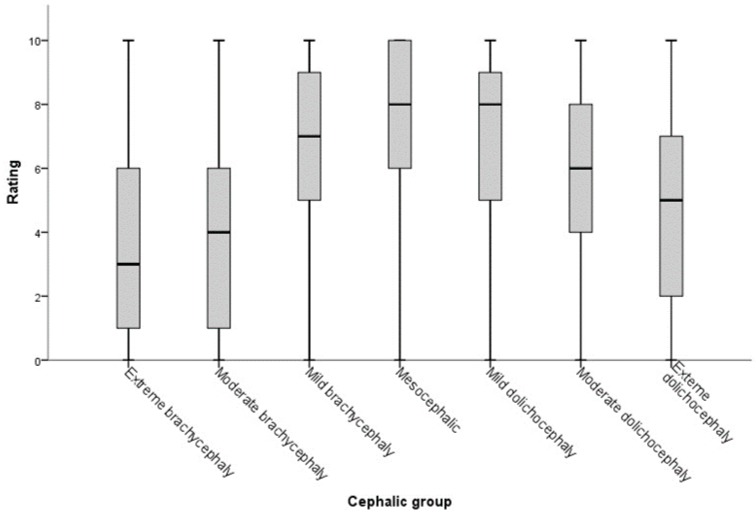
Preference ratings (0 = “I don’t like this cat at all”; 10 = “this is my favorite type of cat”) from 1239 cat owners for *n* = 15 cat images based on cephalic grouping, which was ascribed by expert (*n* = 50) consensus (as per Table 1). Cephalic groupings range from extreme brachycephaly through to extreme dolichocephaly.

Cephalic group was associated with rating score (Kruskal-Wallis statistic (KW) = 4021.9, Degrees of Freedom (df) = 6, *p* < 0.001) with the highest ratings in the mesocephalic group and lowest in the extreme brachycephalic group (Figure 1, Table 2). In univariate analyses, all four phenotypic features considered from the photographs were associated with rating scores (Table 2). The most highly rated phenotypic varieties from each feature were: medium coat length, ginger coat color, green eye color, and mesocephalic skull shape. As there were repeated measures of both cat and owner ID in this dataset that may influence the median scores reported in Table 2, a generalized estimating equation was constructed accounting for these effects and elucidating the underlying preferences.

The generalized estimating equation (Table 3) indicated that four factors were significantly associated with rating score: cephalic grouping, coat length, eye color, and coat color. Cats who exhibited any degree of brachycephaly (extreme, moderate, mild) were rated significantly lower than mesocephalic cats. Similarly, both mild and extreme DC cats were rated significantly lower than mesocephalic cats; however, no difference was found between mesocephalic and moderate DC cats. Cats with medium or long length coats were rated more highly than cats with shorthaired coats and cats with blue-grey, ginger or tabby coats were rated more highly than cats with white/pale/point coats. Finally, cats with blue or green eyes were rated more highly than cats with orange or brown eyes.

### 3.4. Factors Associated with BC Cat Ratings

**Figure 2:**
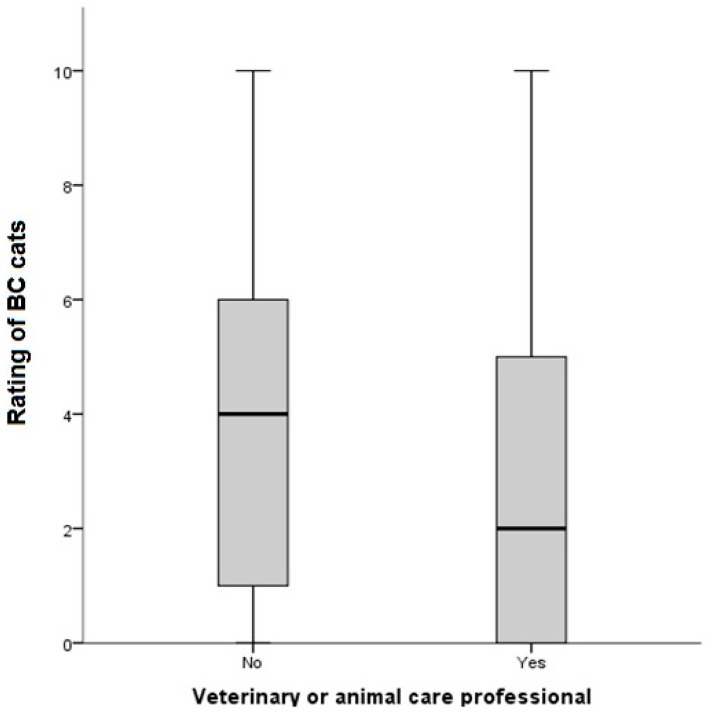
Significant differences (KW = 277.9, df = 2, *p* < 0.001) in ratings of brachycephalic (BC) cat images (*n* = 7/15; as per Table 1) between respondents that identified as veterinary or animal care professionals (*n* = 244) as opposed to those that did not (*n* = 995).

**Figure 3:**
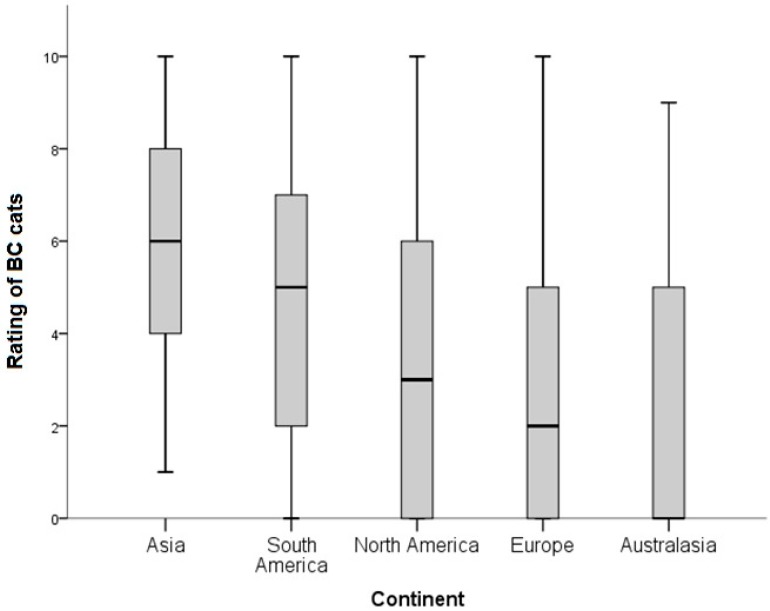
Differences in ratings (0 = I don’t like this cat at all; 10 = This is my favorite kind of cat) for cats identified as having mild to extreme brachycephaly (BC; *n* = 7/15; cat images as per Table 1). Ratings were provided by respondents (*n* = 1239) to two questionnaires. Comparisons are between different continental regions.

**Figure 4:**
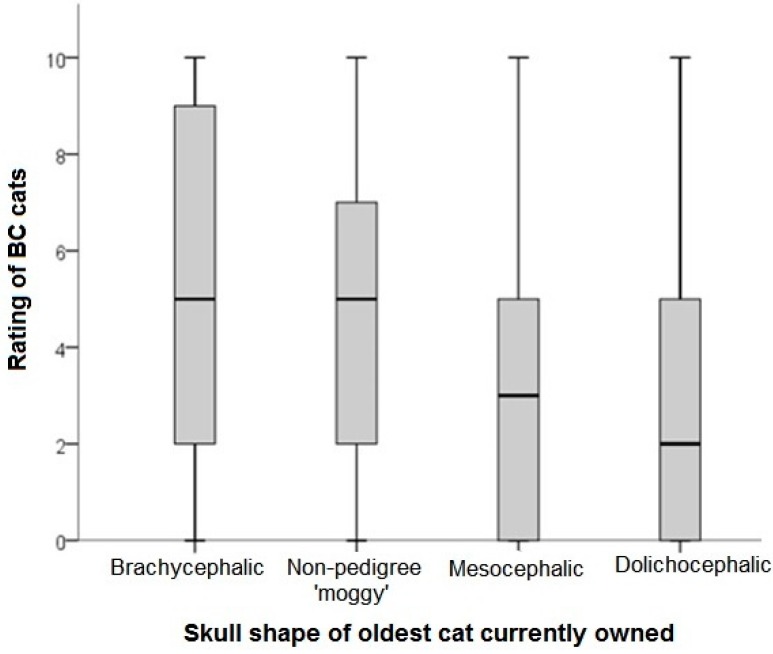
Ratings of liking (0 = I don’t like this cat at all; 10 = This is my favorite kind of cat) for cats identified as having mild to extreme brachycephaly (BC; n = 7/15 cat images as per Table 1). Ratings were provided by *n* = 1239 respondents. Comparisons are relative to skull morphology of the eldest cat currently owned, as based on reported breed and associated pedigree breed standards.

When considering the sub-population of BC cats presented to owners (those cats with a median consensus rating of 1–3, Table 1), several factors were associated with the level of preference of these faces. Those owners who worked in the veterinary or animal care professions rated BC cats significantly lower than those that did not (KW = 277.9, df = 2, *p* < 0.001) (Figure 2). There were significant differences in ratings of BC cats geographically, with respondents living in Asia rating BC cats most highly (KW = 1310.0, df = 4, *p* < 0.001) (Figure 3). Respondents who currently owned BC cats rated BC cats most highly 5 (2–9) (median rating (25th–75th percentiles) as compared with DC owners 2 (0–5); Mesocephalic cat owners: 3 (0–5); and Non-pedigree owners: 3 (0–5); KW = 429.70, *p* < 0.001) (Figure 4). When modelled in a generalized estimating equation with owner and cat picture ID incorporated as random effects, all three variables remained significant (Table 4).

### 3.5. Factors Associated with DC Cat Ratings

**Figure 5:**
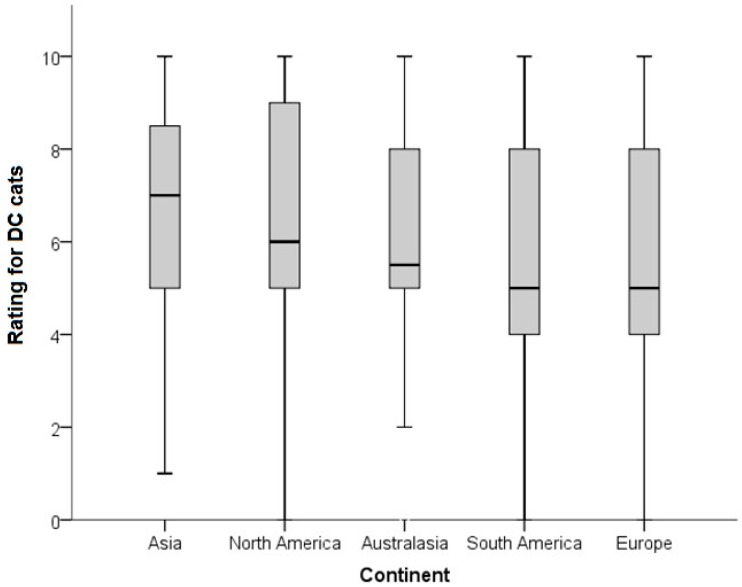
Ratings of liking (0 = I don’t like this cat at all; 10 = This is my favorite kind of cat) for cats identified as having mild to extreme dolichocephaly (DC; *n* = 4/15 cat images as per Table 1). Ratings were provided by respondents (*n* = 1239) to two questionnaires. Comparisons are between different continental regions.

**Figure 6:**
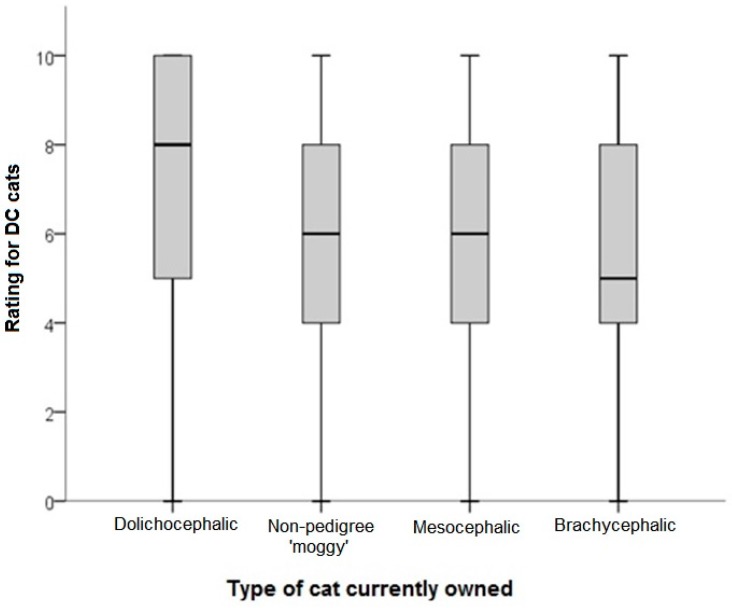
Ratings of liking (0 = I don’t like this cat at all; 10 = This is my favorite kind of cat) for cats identified as having mild to extreme dolichocephaly (DC; *n* = 4/15 cat images as per Table 1). Ratings were provided by respondents (*n* = 1239) to two questionnaires. Comparisons are relative to skull morphology of the oldest cat currently owned, as based on reported breed of oldest owned cat and the associated breed standard.

When considering the sub-population of DC cat images presented to raters (those cats with a median consensus rating of 5–7, Table 1), several factors were associated with the level of preference of these faces. There was no significant difference in the rating of DC cats between respondents who worked in veterinary or animal care professions, as compared to those that did not (MW = 1296765.0, df = 1, *p* = 0.430). There were significant differences in the ratings of DC cat images based on geographical location, with owners who lived in Asia rating DC cats most highly (KW = 48.44, df = 4, *p* < 0.001) (Figure 5). Respondents who owned DC cats rated DC cats most highly 8 (5–8) (median rating (25th–75th percentiles) when compared to other owners: BC owner: 5 (4–8); mesocephalic cat owner: 6 (4–8); non-pedigree owner: 6 (4–8); (KW = 70.62, *p* < 0.001) (Figure 6).

When modelled in a generalized estimating equation with owner and cat ID incorporated as random effects, two variables remained significant, continent and type of cat previously owned (Table 5).

## 4. Discussion

There is a relative paucity of information surrounding preferences for phenotypic features in cats. Research in this area tends to focus on direct acquisition of individuals from shelters [10,11,12]. The current study focused on direct assessments of preference based only on appearance in a static image. It therefore excludes wider factors that may influence preference. These include temperament, human directed behaviors [12], the individual cat’s sex and age [11], and more unusual effects such as providing the individual with a name [10]. Clear differences in preference ratings for skull morphology were observed in this study, with deviations from moderate conformation (mesocephaly) receiving lower preference ratings, which further declined as conformation became more extreme. Mild deviations from mesocephaly were not substantially less preferred in either category (DC or BC), and this may present a mechanism by which more extreme deviations begin to be selected for. The decline in preference appears more marked for BC cats as opposed to DC cats. Previous research suggests that “exotic” cats may be adopted more rapidly from a shelter environment [11]; however, the amalgamated category used in that research included both BC (Persian) and non-BC (ragdoll and Russian blue) breeds. Another study demonstrated that Persian cats had greater odds of adoption from a shelter environment, possibly indicating increased adopter preferences, although only with comparison to domestic short, long and medium coated individuals [10]. Our findings indicate that preferences scored from a static image may differ from broader adopter preferences when direct interaction and ownership intent are involved. The decline in preference for BC cats was greater than that for DC cats, even though the proportion of BC cat owners was larger within the overall sample. This suggests that changes in preference across the spectrum of skull shapes are not simply driven by the proportion of BC/DC owners within the sample.

Brachycephalic cats were given a significantly lower preference rating by veterinary or animal care professionals as opposed to other respondents, an association not seen for DC cats. This reduction in preference rating for extreme BC conformation is more marked within the animal care subsample than within the sample overall. It is likely driven by exposure to the management of medical issues directly associated with brachycephaly in cats [25,29] and knowledge of the general literature surrounding the implications of brachycephaly for breed health e.g., respiratory [19,20,21] and ophthalmic health [23]. Several clinical case reports [30] indicate that brachycephaly has a substantial negative impact on the health of affected cat breeds. The general paucity of studies citing similar health impairments directly associated with dolichocephaly in cats may explain the differences between preferences for extreme DC and BC individuals. Research around breed issues in dogs identifies substantial negative perceptions of BC dogs amongst veterinary professionals, which may be mirrored in this result [36]. Further research is warranted to explore public and professional knowledge and attitudes concerning the impact of brachycephaly and dolichocephaly on cats.

This research is one of the first reports to explore preferences for feline appearance based on geographical location. Respondents residing in Asia were significantly more likely to provide a higher score for both BC and DC cats when compared to respondents from other countries or regions. These preferences were not driven by a greater representation of BC and DC breeds within the Asian sample; of the Asian respondents, none owned a DC breed whilst BC breed ownership was in line with the wider sample. This indicates that preferences for altered skull morphology within Asia may be driven by other, more culturally embedded, factors. Breeding for brachycephaly has a long history, with dog breeds such as the Pug and Pekingese originating from China [37], and BC and DC cat breeds such as the Persian and Siamese originating, or perceived as originating, from the near east [38]. Proximity of breed origin may have an impact on cultural preference and further research is required to explore this dynamic.

Ownership of a particular type of cat was strongly associated with expressed preferences. Respondents whose eldest cat was either BC or DC showed significantly greater preferences for cats that display similar skull morphologies. This was also linked to a substantially diminished preference for cats in the opposite skull shape category (i.e., DC vs. BC and vice versa). The literature suggests that positive “brand” preferences are strongly influenced by prior experiences and knowledge [39]. Likewise, reduced preference for a specific object or idea may be driven by its deviation from something with which the rater has had positive prior experience. It is reasonable to postulate that these effects may apply to specific companion cat breeds. Respondents familiar with, and attached to, a cat of one type (e.g., one with extreme BC) rate similar attributes in other individuals more highly. Conversely, they may also provide a lower rating for individuals with the opposing attribute (e.g., extreme DC). The current study did not explore other issues that may guide preference ratings, such as the ownership of other animals (e.g., dogs) or other cats (both living or deceased) with BC and DC profiles.

As well as general variation around preferences for BC and DC cats, there were also significant differences around coat length, eye color and coat color, although these had a lesser effect as compared to skull shape. These parameters were not assessed relative to the currently or previously owned cat, so they were unable to be linked with prior experience. They were also not evenly represented across the photographs included but were considered important confounding variables and so were included in the analyses. In our study, the reduced preference for white/pale/point cats is not supported by other studies [11], where pale pelages were associated with more rapid adoption. It is also difficult to extricate these preferences from wider perception-based discrimination without more in-depth exploration of respondent’s beliefs—for example, the tendency for people to assign positive or negative traits to orange and white cats, respectively [40]. Similarly, breed characteristics as they are often linked (i.e., extreme DC cats and BC cats are limited to a few breeds such as Siamese and Oriental [DC] or exotic and Persian [BC], which have characteristic features associated with coat color and coat length), making it hard to differentiate the effects from one another. As such, we suggest that the results and conclusions around these variables be treated with caution. Further research is required, using a controlled sample, to explore exactly how and why these phenotypic variations influence preference.

This study is not without its limitations. The number of images was relatively small, and the distribution of BC and DC individuals within the sample was not equivalent. Likewise, the images had a number of poses and backgrounds that likely affected upon the rating. Finally, it is important to acknowledge that the survey content comprised a number of questions about the health of BC cats, which may have negatively affected preference scores for BC images in the immediate term. This said, the authors consider the findings, and their novelty, important in guiding future research and ideas concerning preferences for cats with varying characteristics, including skull shape. This may be especially important considering the current increase in the use of images of cats in social media.

## 5. Conclusions

This work provides novel preliminary data for an, as yet, underexplored phenomenon in the selection of cat breeds by owners. It provides evidence that cranial conformation has a substantial impact when considered alongside other normal variations in a cat’s features, such as eye color and coat color and length, and affects owner preference. There is evidence that both country of residence, profession and ownership experience have a significant impact upon cat owner preference ratings. These parameters may prove useful for future research into the sharing of static images of cats in social networks and the impact this has on decision-making. It may also allow for the development of targeted education regarding breed-associated issues, particularly those linked to skull shape.

## Figures and Tables

**Table 1:** Cephalic rating provided for 15 cat images by veterinarian members of the International Society for Feline Medicine list serve (*n* = 45–50). Ratings were based on the following numerical scale: 1 = extreme brachycephaly (BC) (having the shortest muzzle possible); 2 = moderate BC; 3 = mild BC; 4 = mesocephalic; 5 = mild dolichocephaly (DC); 6 = moderate DC; 7 = extreme DC (having the longest muzzle possible).

Cat Number	Number of Responses	Mean Rating (1–7)	Median Rating (1–7)	Modal Rating (1–7)	Max Rating (1–7)	Min Rating (1–7)
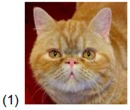	50	1.6	2	2	2	1
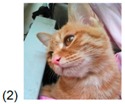	50	4.3	4	4	6	4
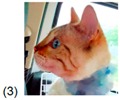	50	4.8	5	5	7	3
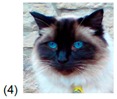	50	3.8	4	4	6	3
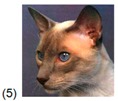	50	5.7	6	6	7	5
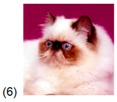	50	1.3	1	1	2	1
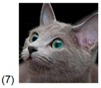	50	4.6	4	4	7	3
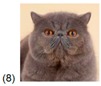	50	1.1	1	1	2	1
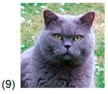	50	3.0	3	3	4	2
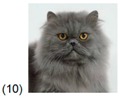 *	47	1.9	2	2	3	1
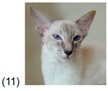 *	47	6.6	7	7	7	5
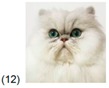 *	47	1.4	1	1	2	1
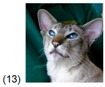 *	46	6.2	6	6	7	4
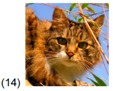 *	46	4.1	4	4	6	3
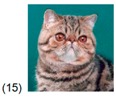 *	45	1.0	1	1	2	1

* These cats were used only in survey two. Those not marked were used in both survey one and two.

**Table 2:** Results of univariate analyses of associations between physical characteristics of 15 cats presented within two questionnaires as photographs (as per Table 1) and owner (*n* = 1239) preference ratings (0 = “I don’t like this cat at all”; 5 = neutral; 10 = “this is my favorite type of cat”).

Variable	Sub-Category	Median Rating	25th–75th Percentile Rating	Kruskal-Wallis Statistic	Kruskal-Wallis Statistic	*p*-Value
Coat length	Long	5	2–7	506.8	<0.001
	Medium	7	5–9		
	Short	6	3–8		
Coat colour	Blue grey	6	3–8	256.7	<0.001
	Ginger	6	4–9		
	Tabby	5	2–9		
	White/Pale/Point	4	1–7		
Eye colour	Blue	5	1–8	348.4	<0.001
	Green	6	4–9		
	Orange or Brown	5	2–8		
Cephalic group	Extreme brachycephaly	3	1–6	4021.9	<0.001
	Moderate brachycephaly	4	1–8		
	Mild brachycephaly	7	5–9		
	Mesocephaly	8	6–10		
	Mild dolichocephaly	8	5–9		
	Moderate dolichocephaly	6	4–8		
	Extreme dolichocephaly	5	2–7		

**Table 3:** Generalized estimating equation of variables that predict respondent’s (*n* = 1239) ratings of cat images (*n* = 15; as per Table 1) based on physical appearance, taking owner and cat identification into account as random effects. BC = Brachycephaly; DC = Dolichocephaly. “Reference” is the sub-category against which those within the same variable were compared.

Variable	Sub-Category	Regression Statistic (B)	Standard Error (SE)	95% Confidence interval (CI)	Wald	*p*
Intercept	-	1.93	0.44	1.05–2.80	18.89	<0.001
Cephalic group	Extreme BC	−2.99	0.12	−3.22–−2.76	649.47	<0.001
	Moderate BC	−4.59	0.12	−4.82–−4.37	1541.88	<0.001
	Mild BC	−1.03	0.09	−1.22–−0.83	106.36	<0.001
	Mesocephalic	Reference				
	Mild DC	−4.63	0.39	−5.39–−3.87	143.60	<0.001
	Moderate DC	0.31	0.21	−0.10–0.72	2.15	0.142
	Extreme DC	−0.87	0.23	−1.32–−0.44	15.18	<0.001
Coat length	Long	3.48	0.21	3.07–3.89	272.03	<0.001
	Medium	1.63	0.16	1.31–1.95	101.25	<0.001
	Short	Reference				
Eye color	Blue	3.63	0.36	2.94–4.33	104.17	<0.001
	Green	1.85	0.17	1.52–2.18	123.12	<0.001
	Orange or Brown	Reference				
Coat color	Blue grey	3.76	0.31	3.14–4.37	143.59	<0.001
	Ginger	6.34	0.46	5.43–7.24	188.33	<0.001
	Tabby	4.61	0.38	3.89–5.33	157.10	<0.001
	White/pale/point	Reference				

**Table 4:** Generalized estimating equation of variables that predict the ratings of cats considered to be brachycephalic (*n* = 7/15 cat images as per Table 1). The group included cats considered to be mildly, moderately or extremely brachycephalic as scored by a panel of veterinarians (*n* = 50; see Table 1). Analyses were conducted taking owner and cat photograph identification (1–15) into account as repeated measures. “Reference” is the sub-category against which those within the same variable were compared. Currently reported cat-type was designated to one of four categories (brachycephalic (BC), mesocephalic, dolichocephalic (DC) or non-pedigree) based on the reported breed of the respondent’s oldest cat.

Variable	Sub-Category	Regression Statistic (B)	Standard Error (SE)	95% Confidence Interval (CI)	Wald	*p*
Intercept	-	5.81	0.61	5.69–5.93	9072.47	<0.001
Animal care profession	No	0.70	0.08	0.54–0.87	71.33	<0.001
	Yes	Reference				
Continent	Asia	0.86	0.15	0.57–1.43	34.02	<0.001
	Australasia	−2.31	0.29	−2.88–−1.74	62.50	<0.001
	North America	−0.89	0.17	−1.22–−0.56	27.79	<0.001
	Europe	−1.73	0.14	−2.01–−1.45	144.74	<0.001
	South America	Reference				
Currently reported cat type	BC	1.18	0.11	0.97–1.40	112.72	<0.001
	DC	−0.56	0.16	−0.89–−0.23	11.31	0.001
	Mesocephalic	−0.19	0.78	−0.35–−0.41	6.18	0.013
	Non-pedigree	Reference				

**Table 5:** Generalized estimating equation of variables that predict the ratings of cats considered to be brachycephalic (*n* = 4/15 cat images as per Table 1). The group included cats considered to be mildly, moderately or extremely dolichocephalic as scored by a panel of veterinarians (*n* = 50; see Table 1). Analyses were conducted taking owner and cat identification into account as repeated measures. “Reference” is the sub-category against which those within the same variable were compared. Currently reported cat type was designated to one of four categories (brachycephalic (BC), mesocephalic, dolichocephalic (DC) or non-pedigree) based on reported breed of the respondent’s oldest owned cat.

Variable	Sub-Category	Regression Statistic (B)	Standard Error (SE)	95% Confidence Interval (CI)	Wald	*p*
Intercept	-	5.69	0.17	5.35–6.02	1104.30	<0.001
Continent	Asia	1.01	0.20	0.61–1.41	24.63	<0.001
	Australasia	0.03	0.33	−0.61–0.68	0.009	0.924
	North America	0.28	0.21	−0.12–0.69	1.87	0.172
	Europe	−0.11	0.20	0.61–1.41	24.63	<0.001
	South America	*Reference*				
Currently reported cat type	BC	−0.15	0.15	−0.44–0.15	0.98	0.323
	DC	1.75	0.21	1.34–2.16	69.87	<0.001
	Mesocephalic	0.15	0.11	−0.06–0.36	1.89	0.169
	Non-pedigree	*Reference*				

## Supplementary Materials

The following are available online at http://www.mdpi.com/2076-2615/8/2/30/s1, Cat Life Style and Face Shape Survey.
